# Comparison of the Ratio of Neck Circumference to Thyromental Distance and the Ratio of Neck Circumference to Inter-Incisor Gap in Predicting Difficult Intubation

**DOI:** 10.7759/cureus.87779

**Published:** 2025-07-12

**Authors:** Lasya Raveendran, Satyajeet Singh, Akash Verma, Manjunatha Chandrashekar

**Affiliations:** 1 Department of Anaesthesiology, Employees' State Insurance Corporation (ESIC) Medical College and Hospital, Hyderabad, IND; 2 Department of Anaesthesiology, Jai Prakash District Hospital, Bhopal, IND; 3 Department of Anaesthesiology, Atal Bihari Vajpayee Government Medical College, Vidisha, IND

**Keywords:** airway assessment, inter-incisor gap, intubation difficulty scale, neck circumference, thyromental distance

## Abstract

Background

Airway management is central to anaesthetic practice, with endotracheal intubation pivotal in both elective and emergency settings. Although various assessment tests aim to predict a difficult airway, their predictive performance varies widely. Comparative evaluation is therefore required to identify the most reliable predictor.

Objective

The study was conducted to compare the two index ratios, the ratio of neck circumference to thyromental distance (RNTMD) and the ratio of neck circumference to inter-incisor gap (RNCIIG), to determine their effectiveness in predicting difficult intubation.

Methods

This prospective observational study consisted of 118 participants undergoing elective surgeries under general anaesthesia. Measurements of neck circumference, thyromental distance, and inter-incisor gap were recorded preoperatively. RNTMD and RNCIIG were calculated. Ease of intubation was determined using the Intubation Difficulty Scale (IDS). Predictive indices and diagnostic accuracy were calculated for both indices.

Results

Both parameters were comparable in terms of specificity, positive and negative predictive values, and diagnostic accuracy. The area under the curve, as calculated from the receiver operating characteristic (ROC), was 0.80 for RNCIIG and 0.96 for RNTMD. However, RNCIIG was found to be more sensitive (80%) than RNTMD (69%).

Conclusion

We conclude that both airway assessment indices, RNCIIG and RNTMD, are effective predictors of a difficult airway. A ratio of two indices provides superior diagnostic accuracy and reliability than when a single parameter is used alone.

## Introduction

Airway management has always been a major concern for anaesthesiologists worldwide [1]. Most anaesthetic maneuvers are targeted at maintaining oxygenation at the tissue level to prevent hypoxia. Endotracheal intubation becomes pivotal in patient management while delivering general anaesthesia or the management of emergency situations. Therefore, careful airway assessment before the induction of anaesthesia is of utmost importance as poor airway management has been recognized as a serious patient safety concern.

A large number of studies have been conducted to develop reliable predictors for a difficult airway. Many studies have tried to develop some bedside tests that are easy to perform and don’t need special equipment, but all these tests have their limitations.

Most of these studies are based on a single parameter or focused on a single subset of patients, like patients with cervical spondylosis or obesity, etc. The diagnostic accuracy of these screening tests varies in different studies. Moreover, most studies do not provide a "measure" of difficult intubation in patients with difficult laryngoscopy. Also, the majority of studies on difficult laryngoscopy and intubation have been performed on the American or European population. Anthropometrically, Indians are different compared to Americans or Europeans.

There are various measurements like neck circumference (NC), thyromental distance (TMD), inter-incisor gap (IIG), hyomental distance, and ratios of these measurements that are employed to predict difficult airways during pre-op assessment of patients. The predictive ability of these tests, as determined by various studies, is highly variable, and there is no consensus as to which among these is the best preliminary test to predict a difficult airway. Therefore, comparison of these methods is inevitable to find the best one.

We compared two of these well-known tests that have recently attracted attention in studies. We conducted this study in the Indian population to compare the predictive values and accuracy of two ratios, the ratio of neck circumference to thyromental distance (RNTMD) and the ratio of neck circumference to inter-incisor gap (RNCIIG), in an attempt to determine which amongst these is more reliable for predicting unanticipated difficult intubation. The Intubation Difficulty Scale (IDS) has been used as a validated score for rating difficult intubations.

## Materials and methods

This observational study was conducted over a period of one and a half years (January 2020 to July 2021), following approval from the Institutional Ethics Committee of People's College of Medical Sciences and Research Centre (approval number: IEC-2019/41). A total of 118 patients scheduled for surgery under general anaesthesia and meeting the inclusion criteria were enrolled in the study. Eligible participants were aged between 18 and 60 years, of either gender, and classified as American Society of Anesthesiologists (ASA) physical status I or II. Patients with ASA grade III or above, cases initially planned for regional anaesthesia that were converted to general anaesthesia intraoperatively, as well as those with oropharyngeal masses, temporomandibular joint immobility, cervical joint instability, or pregnancy, were excluded. Written informed consent was obtained from all study participants.

During the pre-anaesthetic check-up, airway assessment variables, including NC, IIG, and TMD, were measured in the preoperative room one day prior to surgery using a measuring tape and scale. The measurements were taken with the patient sitting upright. NC was measured using a measuring tape encircling the neck just below the laryngeal prominence (Adam's apple) with the head held in a neutral position. TMD was measured as the distance between the thyroid notch and the mentum (chin) with the patient's head fully extended and mouth closed. IIG was measured as the maximum distance between the upper and lower incisor teeth with the mouth open as wide as possible. Both TMD and IIG were measured using a scale. The RNCIIG and RNTMD were then calculated and recorded by an independent anaesthetist who was blinded to the study.

On the day of surgery, patients were transferred to the operation theatre and standard ASA monitors were applied. Intravenous (IV) access was secured, and baseline vitals were recorded. Patients were positioned in the standard “sniffing position” and premedicated with IV midazolam (0.03 mg/kg) and fentanyl (1.5 µg/kg). Following preoxygenation with 100% oxygen for three minutes, anaesthesia was induced with propofol (2 mg/kg). Neuromuscular blockade was achieved using succinylcholine (1.5 mg/kg). After adequate muscle relaxation, laryngoscopy was performed using a Macintosh curved blade laryngoscope by an experienced anaesthesiologist blinded to the study. The difficulty of intubation was assessed by the anaesthesiologist performing intubation, who subsequently completed the IDS [2]. The IDS consists of seven variables (N1-N7) (N1: number of additional intubation attempts; N2: number of additional operators; N3: number of alternative intubation techniques used; N4: laryngoscopic view as defined by the Cormack and Lehane classification; N5: lifting force applied during laryngoscopy; N6: need to apply external laryngeal pressure; N7: position of the vocal cords during intubation (abducted or adducted)). The sum of these variables yielded the final IDS score. An IDS score of 5 or more (IDS >5) was considered indicative of difficult intubation, while an IDS less than 5 (IDS <5) was considered easy intubation.

Statistical analysis

All data analysis was performed using IBM SPSS Statistics for Windows, Version 21 (Released 2013; IBM Corp., Armonk, New York, United States). Frequency distribution and cross-tabulation were conducted to prepare the tables. Quantitative data were expressed as mean and standard deviation, whereas categorical data were presented as numbers and percentages. The chi-square test was performed to obtain the p-value for categorical variables. An independent sample t-test was used to compare the means. Sensitivity, specificity, positive predictive value (PPV), and negative predictive value (NPV) were calculated using standard formulae. The receiver operating characteristic (ROC) curve was employed to identify predictive variables for different parameters, and the area under the curve (AUC) was calculated from the respective ROC curves. A p-value of <0.05 was considered statistically significant.

## Results

Demographic variables such as age and gender were similar between the groups (Table 1).

**Table 1:** Comparison of gender distribution with easy and difficult intubation

Intubation	Gender		Total, n (%)	P-value
	Female, n (%)	Male, n (%)		
Difficult	5 (50)	5 (50)	10 (100)	0.842
Easy	52 (48.15)	56 (51.85)	108 (100)	
Grand total	57 (48.3)	61 (51.7)	118 (100)	

Patients with difficult intubation had a larger NC (38.45 ± 3.69 cm) compared to those with easy intubation (36.58 ± 2.30 cm), with a statistically significant p-value of 0.001. The mean TMD was similar in both groups, with patients with difficult intubation having a mean TMD of 7.41 cm, compared to 7.39 cm in those with easy intubation (Table 2). Patients with difficult intubation had a larger IIG (5.51 ± 0.80 cm) compared to those with easy intubation (5.29 ± 0.62 cm), as indicated by the statistically significant p-value of 0.001 (Table 2).

**Table 2:** Comparison of various airway assessment tests IIG: inter-incisor gap; NC: neck circumference; NPV: negative predictive value; PPV: positive predictive value; RNCIIG: ratio of neck circumference to inter-incisor gap; RNTMD: ratio of neck circumference to thyromental distance; TMD: thyromental distance

Indicators	RNCIIG (≥9.5)	RNTMD (>4.6)	NC (≥41 cm)	IIG (<5 cm)	TMD (≤7.0 cm)
True positive	8	9	5	4	4
True negative	104	100	104	86	52
False positive	4	4	5	6	56
False negative	2	4	4	22	6
Sensitivity	80.00% (44.39% to 97.48%)	69.23% (38.57% to 90.91%)	55.56% (21.20% to 86.30%)	15.38% (4.36% to 34.87%)	40% (12.16% to 7.76%)
Specificity	96.30% (90.79% to 98.98%)	96.15% (90.44% to 98.94%)	95.41% (89.62% to 98.49%)	93.48% (86.34% to 97.57%)	48.15% (38.43% to 57.97%)
PPV	66.67% (42.14% to 84.60%)	69.23% (44.62% to 86.27%)	50.00% (26.18% to 73.82%)	40.00% (16.89% to 68.62%)	6.67% (3.17% to 13.49%)
NPV	98.11% (93.77% to 99.45%)	96.15% (91.70% to 98.26%)	96.30% (92.60% to 98.18%)	79.63% (76.69% to 82.29%)	89.66% (83.44% to 93.71%)
Diagnostic accuracy	94.92% (89.26% to 98.11%)	93.16% (86.97% to 97.00%)	92.37% (86.01% to 96.45%)	76.27% (67.56% to 83.62%)	47.46% (38.19% to 56.85%)

A total of 90% of patients with difficult intubation and 3.7% of patients with easy intubation had an RNTMD > 4.6. Patients with difficult intubation had significantly higher mean RNTMD (5.35 ± 1.24) compared to those with easy intubation (5.06 ± 0.85).

Patients with difficult intubation had a mean RNCIIG of 7.15 while those with easy intubation had a mean RNCIIG of 7.02. Around 80% patients with difficult intubation had an RNCIIG > 9.5.

RNTMD was found to have a sensitivity of 69.23%, specificity of 96.15%, PPV of 69.23%, NPV of 96.15% and diagnostic accuracy of 93.16% (Table 2). The AUC obtained was 0.96, the highest among all airway assessment tests (Table 3). RNCIIG was found to have a sensitivity of 80.00%, specificity of 96.30%, PPV of 66.67%, NPV of 98.11% and a diagnostic accuracy of 94.92% (Table 2). The ROC had an AUC of 0.80 (Table 3).

**Table 3:** Area under the curve (AUC) (calculated from ROC) of various airway assessment tests NC: neck circumference; TMD: thyromental distance; IIG: inter-incisor gap; RNCIIG: ratio of neck circumference to inter-incisor gap; RNTMD: ratio of neck circumference to thyromental distance; ROC: receiver operating characteristic curve

Indicator	AUC	Standard error	P-value	Asymptotic 95% confidence interval	
				Lower bound	Upper bound
NC	0.792	0.085	0.002	0.625	0.958
TMD	0.516	0.102	0.866	0.316	0.716
IIG	0.481	0.122	0.843	0.242	0.720
RNCIIG	0.804	0.097	0.002	0.614	0.995
RNTMD	0.969	0.024	<0.001	0.923	1.000

## Discussion

Airway assessment tests are imperative in the practice of anaesthesia as unanticipated difficult tracheal intubation that can cause intubation delay or failure, significantly increases the morbidity and mortality associated with general anaesthesia [3]. The incidence of difficult laryngoscopy and intubation in various settings has been reported in a wide range of studies, from 1% to 15% [4-9]. In the Indian population, the incidence of difficult laryngoscopy and intubation was 9.7% and 4.5% respectively, according to Prakash et al. [10]. Several indices, including the Mallampati score, TMD, IIG, and NC, have been employed to assess difficult airways. However, these tests are often criticized for their limited predictive accuracy. Consequently, combining such parameters, particularly in ratio form, has demonstrated improved outcomes. This study aims to compare the efficacy of the RNTMD and RNCIIG ratios in predicting difficult airways.

According to the data analysed, it was found that cases of difficult intubation were evenly distributed in various age groups. There was no statistical significance between age and the incidence of difficult intubation, as determined by an insignificant p-value (p = 0.6). The finding is contrary to studies by Han et al. [11] and Karakus et al. [12], where older patients were found to have a greater incidence of difficult intubation as compared to younger patients. No such observation was found in our study.

The gender distribution in the study population was nearly even, with a mild male preponderance (with 61 males and 57 females). The incidence of difficult intubation in both genders was found to be similar. The incidence was 8.7% in females and 8.1% in males. The finding is contrary to studies by Karkouti et al. [13], where a male preponderance was seen in difficult intubation cases. The incidence of difficult intubation was 13.6% in males and 5% in females.

The ASA distribution in both groups of easy and difficult intubation was also statistically insignificant, as ascertained by an insignificant p-value (p = 0.992). A study by Manayaliul [14] found an increased incidence of difficult intubation in ASA classes II and III patients (73%). They had a cohort of obese patients with comorbidities in order of hypertension, diabetes mellitus, and hypothyroidism. Our study, however, included patients with ASA physical status I and II as the study population, and no significant change in the incidence of difficult intubation was noted in either group. In other words, age, gender, and ASA grade were found to be statistically insignificant, therefore inconsequential in predicting difficult intubation cases.

In a study by Han et al., RNTMD was found to be the most effective airway assessment tool, having a sensitivity of 80% and a specificity of 62.9%. In our study, the sensitivity and specificity were 69.2% and 96%, respectively. Also, the PPV, as per our study, was significantly higher at 69.2% as against 29% in the study by Han et al. NPV was similar, and the AUC obtained was 0.7 as against 0.9 in our study. In the same study, the RNCIIG was found to have a sensitivity of 88.6% and a specificity of 62.9%. The PPV and NPV were 32% and 96.6%, respectively. In our study, we found RNCIIG to have a similar sensitivity and NPV of 80% and 98%, but a much higher PPV of 66.6% and a specificity of 96.3%. The AUC was calculated to be 0.8. The findings of this study closely corroborate with the findings of our study, as this study has ascertained RNCIIG as superior to other indicators, including RNTMD. In our study, we found that though both indices are very good indicators for predicting difficult airway and comparable in most parameters, RNCIIG is superior due to its higher sensitivity. The AUC for both ratios is comparable.

In a study conducted by Manayaliul [14] in obese patients, RNTMD was found to have the best predictive ability as compared to other airway assessment tests. The study, however, did not assess RNCIIG as an indicator. They found RNTMD to have a sensitivity of 76.9%, specificity of 89.4%, PPV and NPV of 65.6% and 93.7%, respectively. The AUC was calculated as 0.85. The findings are comparable to our study, where we found similar results in all parameters.

A study by Kim et al. [15] recruited a cohort of 123 patients and recorded a lower PPV of 45.5% for RNTMD. Other parameters were comparable.

In a study by Hirmanpour et al. [16] involving obstetric patients alone, the sensitivity and specificity of RNTMD were 71.7% and 70%, respectively. Other study parameters included a PPV of 17% and an AUC of 0.68. The findings were in contrast to our study, where we found RNTMD to have fairly good sensitivity and very good specificity, PPV, and NPV. The variation in results could possibly be due to the recruitment of obstetric patients exclusively for the study. Due to increased soft tissue in the neck, proper assessment of the TMD may have been difficult. Our study, however, excluded obstetric patients; therefore, no such confounder was seen. The efficacy of RNTMD in these subsets of patients with a known risk factor for difficult intubation, like obstetric cases, may be evaluated further.

The diagnostic accuracy of these parameters substantially increases when a ratio of two such parameters is used. Hence, we found both RNTMD and RNCIIG to have very good predictive ability as determined by their high AUC and diagnostic accuracy. Both these ratios, therefore, could be used as effective predictors of difficult intubation. When the two ratios are compared, RNCIIG is slightly superior to RNTMD due to its better sensitivity.

Limitations of the study

The participant size in the current study was smaller as compared to similar studies. Complete blinding was not possible as the anaesthesiologist performing intubation could recognize physical characteristics suggestive of difficult intubation. Also, he would perform some airway assessment tests himself prior to intubation. This may also lead to confirmation bias and false reporting of a higher IDS score; for instance, the lifting force required during laryngoscopy may be perceived to be higher.

## Conclusions

We found both RNCIIG and RNTMD to be highly reliable in predicting a difficult airway, as evident from the ROC curves and high diagnostic accuracy of both tests. However, RNCIIG remains slightly superior due to its higher sensitivity. These tests are simple, easy to perform, and require minimal resources or time. When incorporated into routine pre-anaesthetic check-ups, they may significantly reduce the incidence of unanticipated difficult intubations.
